# A moderate spinal contusion injury in rats alters bone turnover both below and above the level of injury with sex-based differences apparent in long-term recovery

**DOI:** 10.1016/j.bonr.2024.101761

**Published:** 2024-04-10

**Authors:** Corinne E. Metzger, Robert C. Moore, Alexander S. Pirkle, Landon Y. Tak, Josephina Rau, Jessica A. Bryan, Alexander Stefanov, Matthew R. Allen, Michelle A. Hook

**Affiliations:** a Department of Anatomy, Cell Biology, and Physiology, Indiana University School of Medicine, Indianapolis, IN, United States of America; b Department of Neuroscience and Experimental Therapeutics, Texas A&M University School of Medicine, Bryan, TX, United States of America

**Keywords:** Spinal cord injury, Sublesional vs. supralesional, Bone formation rate, Sex differences

## Abstract

Spinal cord injury (SCI) leads to significant sublesional bone loss and high fracture rates. While loss of mechanical loading plays a significant role in SCI-induced bone loss, animal studies have demonstrated mechanical loading alone does not fully account for loss of bone following SCI. Indeed, we have shown that bone loss occurs below the level of an incomplete moderate contusion SCI, despite the resumption of weight-bearing and stepping. As systemic factors could also impact bone after SCI, bone alterations may also be present in bone sites above the level of injury. To examine this, we assessed bone microarchitecture and bone turnover in the supralesional humerus in male and female rats at two different ages following a moderate contusion injury in both sub-chronic (30 days) and chronic (180 days) time points after injury. At the 30-day timepoint, we found that both young and adult male SCI rats had decrements in trabecular bone volume at the supralesional proximal humerus (PH), while female SCI rats were not different from age-matched shams. At the 180-day timepoint, there were no statistical differences between SCI and sham groups, irrespective of age or sex, at the supralesional proximal humerus. At the 30-day timepoint, all SCI rats had lower BFR and higher osteoclast-covered trabecular surfaces in the proximal humerus compared to age-matched sham groups generally matching the pattern of SCI-induced changes in bone turnover seen in the sublesional proximal tibia. However, at the 180-day timepoint, only male SCI rats had lower BFR at the supralesional proximal humerus while female SCI rats had higher or no different BFR than their age-matched counterparts. Overall, this preclinical study demonstrates that a moderate contusion SCI leads to alterations in bone turnover above the level of injury within 30-days of injury; however male SCI rats maintained lower BFR in the supralesional humerus into long-term recovery. These data further highlight that bone loss after SCI is not driven solely by disuse. Additionally, these data allude to potential systemic factors exerting influence on bone following SCI and highlight the need to consider treatments for SCI-induced bone loss that impact both sublesional and systemic factors.

## 1 Introduction

Spinal cord injury (SCI) leads to significant bone loss resulting in bone fragility and high rates of nontraumatic fracture post-injury (Vestergaard et al., 1998). Fracture rates increase with time post-injury (Zehnder et al., 2004). Clinical studies demonstrate rapid bone loss in the lower limbs (Szollar et al., 1997; Biering-Sørensen et al., 1990; Dauty et al., 2000; Eser et al., 2004; Garland et al., 2001) and the highest fracture rates in the tibia and femur (Vestergaard et al., 1998; Zehnder et al., 2004; Comarr et al., 1962; Gifre et al., 2014; Grassner et al., 2018). Moreover, post-fracture complications are high among people with SCI, leading to an increased burden of care and further reduced quality of life after injury (Gifre et al., 2014; Grassner et al., 2018; Carbone et al., 2013; Sinnott et al., 2022).

Bone loss below the level of injury is expected given the reduced loading of the lower limbs after SCI; however, animal studies have demonstrated that SCI-induced bone loss is greater than bone loss in other models of disuse, including hindlimb immobilization (Liu et al., 2008) or sciatic neurectomy (Jiang et al., 2006). Additionally, previous work from our group has shown lower trabecular bone volume and altered bone turnover at the proximal tibia following a moderate spinal contusion injury, despite weeks to months of regained weightbearing and locomotor function (Metzger et al., 2018; Metzger et al., 2022). These pre-clinical studies clearly demonstrate that bone loss is different and greater than can be explained solely by disuse-related factors, including reduced mechanical loads, and decreased or absent contraction of skeletal muscles. Clinical studies suggest that SCI-related factors that could contribute to bone loss include a pro-inflammatory state (Wang et al., 2007; Gibson et al., 2008; Davies et al., 2007; Hayes et al., 2002) and alterations in endocrine factors (Bauman et al., 2006; Sullivan et al., 2017; Boehl et al., 2022). Animal models have also demonstrated systemic inflammation (Metzger et al., 2022; Maldonado-Bouchard et al., 2016) and hormonal changes following SCI (Otzel et al., 2019; Hubscher et al., 2006; del Rivero and Bethea, 2016). Any systemic factor contributing to SCI-induced bone loss would be expected to have effects on skeletal sites above the injury.

While a majority of clinical studies report bone loss only in the sublesional lower limbs (Zehnder et al., 2004; Biering-Sørensen et al., 1990; Dauty et al., 2000; De Bruin et al., 2005; Ghasem-Zadeh et al., 2021a), there are data to suggest reduced bone in both sublesional and supralesional sites after SCI (Finsen et al., 1992; Sabo et al., 2001). Notably, severity in injury, location of injury, and time from injury make conclusions within clinical data challenging. Similarly in animal models, most studies have focused on sublesional bone loss only, and the studies that have included analyses of supralesional bones are contradictory. While Liu et al., found no differences in the humeri of rats 21 days following complete transection of the thoracic spinal cord, compared with age-matched intact controls (Liu et al., 2008), del Rivero and Bethea reported lower total body and humeral bone mineral density in mice four weeks following SCI (del Rivero and Bethea, 2016). While these contradictory data in two different rodent models were collected at approximately the same stage of injury, in sub-chronic timeframes (21–28 days), subtle differences in timing, interactions between age, sex and injury, or even species differences may contribute to the differences across studies. Any impact of systemic factors alone on supralesional bones after SCI may also take longer to manifest, compared with sublesional bone loss that would be rapidly impacted by interactions with mechanical unloading.

Previously, we assessed bone structural parameters and bone turnover in the sublesional tibia after a moderate contusion injury at sub-chronic and chronic timepoints in male and female rats at two different ages (Metzger et al., 2022). Even at the chronic timepoint, months after resumption of regular weightbearing, SCI rats in most groups had lasting decrements in tibial trabecular microarchitecture compared to age-matched controls. The goal of this current study was to assess the supralesional humerus within these sub-chronic and chronic recovery timepoints, to determine if there are differences in sublesional and supralesional bone sites following a moderate contusion injury in rats. We hypothesized that the sublesional tibia would have greater changes in bone microarchitecture and bone turnover compared to sham-treated controls, while the supralesional humerus would be minimally impacted by a lower thoracic SCI.

## 2 Methods

### 2.1 Animals

Animals from this study are a subset of rats previously described (Metzger et al., 2022). Proximal tibia, proximal humerus, 4th lumbar vertebra from eight different sets of spinal cord injured and age-matched sham rodents were examined: young male and female and adult male and female at both 30 days and 180 days post injury (n = 6–8/group). Young (3 months) and adult (9 months) male and female Sprague-Dawley rats (Envigo, Houston, TX, USA) were utilized from the parent protocol. As previously described, all rats were acclimated to the vivarium at Texas A&M Health Science Center for 14 days prior to study initiation. Rats were individually housed in Plexiglas cages [45.7 (length) x 23.5 (width) x 20.3 (height) cm], with their positions on the cage racks randomized across experimental groups. Rats had free access to food and water. Housing rooms were maintained on a fixed 12-h light/dark cycle, with surgeries and behavioral tests conducted during the light cycle. Following the spinal contusion injury, the rats' bladders were expressed manually every morning and evening until they regained bladder function (operationally defined as voiding on own for 3 consecutive days). Fluorochrome calcein injections were given to all rats 9 and 2 days prior to euthanasia for bone formation rate analyses. At 30 day or 180 days, rats were humanely euthanized with a lethal overdose of pentobarbitol (Fatal Plus, 100 mg/kg). All experiments were reviewed and approved by the Institutional Animal Care Committee at Texas A&M prior to initiating the studies and were consistent with the NIH guidelines for animal care and use.

### 2.2 Spinal contusion injury

Rats were given a moderate spinal contusion (T11-T12) or a sham injury. The moderate contusion injury was produced by applying an impact force (150 kdynes with a 1 s dwell time) onto the T11-T12 spinal tissue with an Infinite Horizon (IH) spinal cord impactor (PSI, Fairfax Station, VA, USA), as previously described (Metzger et al., 2022). Sham rats were treated identically to the SCI rats with the same laminectomy, but the impact force was not applied to the spinal cord tissue and the contusion injury was not produced. As described previously, rats were treated with 100,000 units/kg Pfizerpen (penicillin G potassium) immediately after surgery and again 2 days later to prevent infection. For the 24-h post-surgery, rats were placed in a recovery room maintained at 26.6 °C. To compensate for fluid loss, subjects were given 3 ml of saline after surgery. Michel clips were removed 14 days after surgery (Aceves, 2019). Assessment of locomotor recovery with the Basso, Beattie and Bresnahan (BBB) scale is previously reported, but all rats assessed here regained locomotor function. No age effects were found for males in recovery of locomotor function, but the middle-aged adult females had lower locomotor recovery than the young females (Metzger et al., 2022).

### 2.3 Micro-computed tomography

All excised bones were fixed in 1 % phosphate-buffered formalin for ∼24 h and subsequently stored in 70 % ethanol. All bone samples were shipped to Indiana University School of Medicine for subsequent processing and analysis. The proximal tibia and the proximal humerus were scanned on a SkyScan 1172 (Bruker, Billerica, MA, USA) with a 0.5 mm aluminum filter and a 12-μm voxel size. The 4th lumbar vertebra was scanned on a SkyScan 1176 (Bruker) with a 0.5 mm aluminum filter and 18-μm voxel size. All scans were processed utilizing Bruker software (CTAn). For the proximal tibia and proximal humerus, trabecular bone was analyzed in a 1 mm region beginning approximately 0.5 mm distal to the proximal growth plate. Trabecular bone at the 4th lumbar vertebra was assessed in a 1.5 mm region ∼0.6 mm from the caudal end of the bone. Cortical bone was analyzed as an average of 5 contiguous slices 4 mm below the end of the trabecular region of interest in the proximal tibia and from 5 contiguous slices 1 mm below the trabecular region in the proximal humerus. Trabecular and cortical parameters reported follow the recommended guidelines for rodent bone microarchitectural assessment (Bouxsein et al., 2010). Data from the proximal tibia were previously published in the parent study (Metzger et al., 2022).

### 2.4 Histomorphometry

After μCT scanning, the proximal tibia and proximal humerus were serially dehydrated and embedded in methyl methacrylate (Sigma Aldrich, St. Louis, MO). 4-μm thick frontal sections were obtained and mounted on slides. One slide was left unstained for assessment of fluorochrome calcein labels. A trabecular region of interest excluding primary spongiosa and endocortical surfaces, was utilized to measure total bone surface (BS), single-labeled surface (sLS), double-labeled surface (dLS), and interlabel distances at 20× magnification. Mineralized surface to bone surface (MS/BS; [dLS+(sLS/2)]/BS*100), mineral apposition rate (MAR; average interlabel distance/7 days), and bone formation rate (BFR/BS; [MS/BS*MAR]*3.65) were calculated. An additional slide was stained for tartrate resistant alkaline phosphatase (TRAP) and counter stained with toluidine blue for analysis of osteoclast-covered trabecular surfaces normalized to total trabecular bone surface (Oc.S/BS, %). BIOQUANT (BIOQUANT Image Analysis, Nashville, TN) was used to complete all histomorphometric analyses and analyzed by the same individual. All nomenclature follows standard usage (Dempster et al., 2013). Histomorphometry data from the proximal tibia were previously published in the parent study (Metzger et al., 2022).

### 2.5 Statistical analyses

Each set of data was tested to determine if assumptions for normal distribution were met utilizing Shapiro-Wilk. Normally distributed data were tested with a 2 × 2 factorial ANOVA to assess the main and interaction effects of condition (sham vs. SCI) and time (30d vs. 180d post-injury) within each bone site. If the model p-value was statistically different (p < 0.05), a Tukey HSD test was run to assess pairwise comparisons. If normality assumptions were not met for data, a Kruskal-Wallis test was performed. If the test was statistically different, a Steel-Dwass All Pairs test was completed to assess pairwise comparisons. When non-parametric tests were used, the Kruskal-Wallis is noted in parentheses with the p-value. The main and interaction effects are reported for normally distributed data. All data are presented as box and whiskers plots with all individual data points shown. All statistical analyses were completed on JMP Pro version 17 (SAS, Cary, NC, USA).

## 3 Results

### 3.1 All rats regained hindlimb weight-supported stepping by 15 days post-injury

There was some variation in recovery of weight-supported stepping, with the young females displaying weight supported stepping by Day 9 post injury, the young and middle-aged males by day 11, and the middle-aged females by Day 15. Irrespective of age or sex, all rats had recovered weight-supported stepping on the hindlimbs by Day 15 post injury. On day 15 post SCI, converted BBB scores ranged from 8.56 ± 0.77 (unconverted 10.31 ± 0.89) in the middle-aged female rats to 10.64 ± 0.39 (unconverted BBB 13.13 ± 0.55) in the young male rats. By 30- and 180-days post injury, all rats had unconverted BBB scores >10.1 ± 0.51 and 10.4 ± 0.69, respectively. These data have been previously reported in Metzger, et al. (Metzger et al., 2022)

### 3.2 Young male rats had main effects of condition (SCI vs. Sham) on trabecular bone at both the sublesional tibia and supralesional humerus with lower trabecular bone volume in SCI

**Fig. 1:**
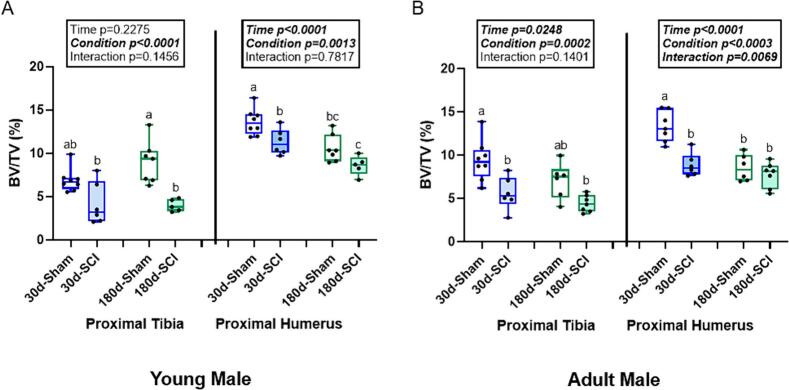
Trabecular bone volume (BV/TV, %) of sham and SCI rats at 30- and 180-days post injury in the proximal tibia (PT) and proximal humerus (PH) in young and adult male rats. A). In young male rats, there was a main effect of condition with 180d-SCI lower than 180d-Sham at the PT. At the PH, there was an effect of time and condition with 30d-SCI lower than 30d-Sham. (B) In the adult males, there was an effect of time and condition at the PT. 30d-SCI was lower than 30d-Sham with no statistical differences between the 180d groups. At the PH, there was an effect of time, condition, and an interaction effect with 30d-SCI lower than 30d-Sham and both 180d groups not different from each other. n = 6–8/group. Groups not sharing the same letter are statistically different from each other within bone site. Main and interaction effect p-values are denoted in the box above the bone site.

Despite rapid recovery of hindlimb weight-bearing, in young male rats, there was a main effect of condition (p = 0.0003) on trabecular bone volume at the proximal tibia, but no effect of time (p = 0.2275) and no interaction effect (p = 0.1456). 180d-Sham rats had higher trabecular bone volume than 180d-SCI while 30d-SCI was lower, but not statistically different from 30d-Sham (Fig. 1). For trabecular thickness, there was a main effect of time (p < 0.0001) and condition (p = 0.0002), but no interaction effect (p = 0.4654). 180d-Sham rats had greater trabecular thickness than all other groups. There was a main effect of condition in trabecular separation (p = 0.0045), but no effect of time (p = 0.9585) and no interaction effect (p = 0.0714). 180d-Sham rats had lower trabecular separation than all other groups. There was a main effect of condition (p = 0.0002) on trabecular number, but no effect of time (p = 0.9967) and no interaction effect (p = 0.2862). 180d-Sham rats had greater trabecular number than both 180d-SCI and 30d-SCI rats but were not statistically different from 30d-Sham rats (Table 1).

**Table 1:** Trabecular bone parameters of the proximal tibia and the proximal humerus. Trabecular thickness (Tb.Th), trabecular separation (Tb.Sp), and trabecular number (Tb.N). Groups not sharing the same letter within sex, age, and bone site are statistically different (p < 0.05).

	Site	Time	Condition	Tb. Th (mm)	Tb. Sp (mm)	Tb. N (mm/#)
Young Male	Tibia	30d	Sham	0.07 ± 0.01^b^	0.42 ± 0.04^a^	0.98 ± 0.17^a^
			Contusion	0.06 ± 0.00^b^	0.45 ± 0.09^a^	0.66 ± 0.32^a^
		180d	Sham	0.08 ± 0.01^a^	0.37 ± 0.05^b^	1.09 ± 0.26^a^
			Contusion	0.07 ± 0.01^a^	0.49 ± 0.03^a^	0.56 ± 0.08^b^
	Humerus	30d	Sham	0.08 ± 0.01^b^	0.37 ± 0.03^b^	1.58 ± 0.18^a^
			Contusion	0.07 ± 0.00^b^	0.41 ± 0.04^a^	1.29 ± 0.16^a^
		180d	Sham	0.11 ± 0.01^a^	0.42 ± 0.09^a^	1.01 ± 0.11^a^
			Contusion	0.11 ± 0.01^a^	0.47 ± 0.12^a^	0.84 ± 0.14^b^
Adult Male	Tibia	30d	Sham	0.08 ± 0.01^a^	0.37 ± 0.03^b^	1.12 ± 0.19^a^
			Contusion	0.07 ± 0.00^b^	0.41 ± 0.04^ab^	0.80 ± 0.24^b^
		180d	Sham	0.09 ± 0.01^a^	0.44 ± 0.03^a^	0.79 ± 0.18^b^
			Contusion	0.07 ± 0.01^b^	0.45 ± 0.05^a^	0.60 ± 0.11^b^
	Humerus	30d	Sham	0.08 ± 0.00^b^	0.42 ± 0.07^b^	1.59 ± 0.22^a^
			Contusion	0.09 ± 0.01^b^	0.57 ± 0.05^a^	1.02 ± 0.16^b^
		180d	Sham	0.12 ± 0.02^a^	0.60 ± 0.06^a^	0.74 ± 0.10^bc^
			Contusion	0.11 ± 0.02^ab^	0.57 ± 0.06^a^	0.72 ± 0.10^c^
Young Female	Tibia	30d	Sham	0.08 ± 0.00^ab^	0.20 ± 0.02^b^	3.31 ± 0.30^a^
			Contusion	0.07 ± 0.01^b^	0.21 ± 0.02^b^	2.86 ± 0.42^a^
		180d	Sham	0.09 ± 0.01^a^	0.45 ± 0.03^a^	1.13 ± 0.14^b^
			Contusion	0.09 ± 0.00^a^	0.55 ± 0.02^a^	0.83 ± 0.22^c^
	Humerus	30d	Sham	0.09 ± 0.00	0.22 ± 0.03^ab^	3.40 ± 0.22
			Contusion	0.08 ± 0.00	0.25 ± 0.05^a^	3.06 ± 0.37
		180d	Sham	0.09 ± 0.01	0.21 ± 0.04^b^	3.37 ± 0.48
			Contusion	0.09 ± 0.01	0.23 ± 0.02^ab^	3.04 ± 0.36
Adult Female	Tibia	30d	Sham	0.11 ± 0.01	0.42 ± 0.09	1.27 ± 0.49
			Contusion	0.11 ± 0.01	0.47 ± 0.12	1.09 ± 0.44
		180d	Sham	0.09 ± 0.01	0.45 ± 0.03	1.13 ± 0.14
			Contusion	0.09 ± 0.01	0.55 ± 0.10	0.83 ± 0.22
	Humerus	30d	Sham	0.10 ± 0.01	0.60 ± 0.10	1.47 ± 0.44
			Contusion	0.10 ± 0.01	0.66 ± 0.04	1.28 ± 0.28
		180d	Sham	0.09 ± 0.01	0.66 ± 0.05	1.35 ± 0.27
			Contusion	0.10 ± 0.01	0.68 ± 0.05	1.12 ± 0.30

At the proximal humerus in young male rats, there was a main effect of both time (p < 0.0001) and condition (p = 0.0013) on trabecular bone volume, but no interaction (p = 0.7817). 30d-SCI had lower trabecular bone volume than 30d-Sham with 180d-SCI lower than both 30d groups, but not statistically lower than 180d-Sham (Fig. 1). Trabecular thickness did not meet the normality assumptions (Kruskal-Wallis p = 0.0003). Both 180d groups had thicker trabecular bone than both 30d groups. There was a main effect of both time (p = 0.0003) and condition (p = 0.0122) for trabecular separation, but there was no interaction effect (p = 0.4579). 30d-Sham rats had lower trabecular separation than all other groups. For trabecular number, there was a main effect of both time (p < 0.0001) and condition (p = 0.0122) but there was no interaction effect (p = 0.3143). 180d-SCI rats had lower trabecular number than all other groups (Table 1).

At 4th lumbar vertebra in young male rats, there was a main effect of condition (p = 0.0068) for trabecular bone volume, but no effect of time (p = 0.0759) and no interaction effect (p = 0.3205). 30d-Sham rats had greater trabecular bone volume than 180d-SCI rats, but were not statistically different from 30d-SCI or 180d-Sham rats. There was a main effect of time (p = 0.0011) and condition (p = 0.0050) for trabecular thickness, but no interaction effect (p = 0.3062). The 180d-Sham group had greater trabecular thickness than all other groups. Trabecular separation did not meet the normality assumptions (Kruskal-Wallis p = 0.0027). 180d-SCI rats had greater trabecular separation than 30d-Sham rats, but were not statistically different from 180d-Sham or 30d-SCI rats. There was a main effect of time (p = 0.0005) and condition (p = 0.0206) for trabecular number, but no interaction effect (p = 0.3916). Both 30d groups had greater trabecular number than 180d-SCI rats but were not statistically different from 180d-Sham rats (Table 2).

**Table 2:** Trabecular bone parameters of the 4th lumbar vertebra. Trabecular bone volume (BV/TV), trabecular thickness (Tb.Th), trabecular separation (Tb.Sp), and trabecular number (Tb.N). Groups not sharing the same letter within sex and age are statistically different (p < 0.05).

	Time	Condition	BV/TV (%)	Tb. Th (mm)	Tb. Sp (mm)	Tb. N (mm/#)
Young Male	30d	Sham	34.88 ± 3.85^a^	0.09 ± 0.00^b^	0.17 ± 0.01^a^	3.89 ± 0.29^a^
		Contusion	31.02 ± 4.18^ab^	0.09 ± 0.01^b^	0.18 ± 0.01^ab^	3.62 ± 0.33^a^
	180d	Sham	33.23 ± 5.39^ab^	0.10 ± 0.01^a^	0.20 ± 0.02^ab^	3.35 ± 0.60^ab^
		Contusion	25.42 ± 5.81^b^	0.09 ± 0.01^b^	0.24 ± 0.03^a^	2.80 ± 0.06^b^
Adult Male	30d	Sham	27.59 ± 5.90	0.08 ± 0.01^ab^	0.20 ± 0.01^b^	3.32 ± 0.29^a^
		Contusion	23.94 ± 4.17	0.08 ± 0.00^b^	0.22 ± 0.04^ab^	3.01 ± 0.46^ab^
	180d	Sham	28.89 ± 2.75	0.10 ± 0.01^a^	0.25 ± 0.02^a^	2.89 ± 0.22^ab^
		Contusion	23.86 ± 5.58	0.09 ± 0.01^ab^	0.25 ± 0.02^a^	2.61 ± 0.40^b^
Young Female	30d	Sham	38.63 ± 1.63^ab^	0.10 ± 0.00^a^	0.18 ± 0.01	3.98 ± 0.19
		Contusion	33.65 ± 3.73^b^	0.09 ± 0.00^b^	0.18 ± 0.02	3.83 ± 0.29
	180d	Sham	43.76 ± 6.45^a^	0.11 ± 0.01^a^	0.16 ± 0.02	4.10 ± 0.29
		Contusion	40.83 ± 4.67^a^	0.10 ± 0.00^a^	0.17 ± 0.02	4.02 ± 0.30
Adult Female	30d	Sham	35.43 ± 6.00	0.11 ± 0.01	0.23 ± 0.03	3.32 ± 0.33
		Contusion	34.77 ± 7.08	0.11 ± 0.01	0.22 ± 0.02	3.27 ± 0.37
	180d	Sham	37.45 ± 4.74	0.11 ± 0.01	0.23 ± 0.02	3.31 ± 0.28
		Contusion	35.02 ± 5.24	0.11 ± 0.01	0.24 ± 0.02	3.10 ± 0.30

### 3.3 Adult male rats also had main effects of condition (SCI vs. Sham) at both sub- and supralesional sites with lower supralesional trabecular bone volume at the 30d timepoint

At the proximal tibia in adult male rats, there was a main effect of both time (p = 0.0248) and condition (p = 0.0002) on trabecular bone volume, but there was no interaction effect (p = 0.4501). 30d-Sham rats had greater trabecular bone volume than both 30d-SCI and 180d-SCI rats but were not statistically different from 180d-Sham rats (Fig. 1). There was a main effect of condition (p < 0.0001) for trabecular thickness, but there was no effect of time (p = 0.0947) or interaction effect (0.7412). Both Sham groups had higher trabecular thickness than both SCI groups. There was a main effect of time (p = 0.0004) for trabecular separation, but no effect of condition (p = 0.1098) and no interaction effect (p = 0.3049). Both 180d-Sham and 180d-SCI rats had greater trabecular separation than 30d-Sham rats but were not statistically different from 30d-SCI rats. There was a main effect of both time (p = 0.0012) and condition (p = 0.0016) for trabecular number, but no interaction effect (p = 0.3543). 30d-Sham rats had greater trabecular number than all other groups (Table 1).

At the proximal humerus in adult male rats, there was a main effect of time (p < 0.0001), condition (p = 0.0003), and an interaction effect (p = 0.0069) for trabecular bone volume. 30d-Sham rats had greater trabecular bone volume than all other groups (Fig. 1). Trabecular thickness did not meet normality assumptions (Kruskal-Wallis p = 0.0031). 180d-Sham rats had thicker trabecular bone than both 30d groups, but were not statistically different from 180d-SCI rats. There was a main effect of time (p = 0.0014), condition (p = 0.0285), and an interaction effect (p = 0.0012) for trabecular separation. 30d-Sham had lower trabecular separation than all other groups. Trabecular number did not meet normality assumptions (Kruskal-Wallis p = 0.0002). 30d-Sham rats had greater trabecular number than 30d-SCI rats and both 180d groups (Table 1).

At the L4 vertebra in adult males, the statistical model did not show any significant differences for trabecular bone volume (p = 0.2022). Trabecular thickness did not meet normality assumptions (p = 0.0261). 180d-Sham rats had higher trabecular thickness than 30d-SCI rats. There was a main effect of time (p = 0.0004) for trabecular separation, but no effect of condition (p = 0.1251) and no interaction effect (p = 0.4674). Both 180d groups had greater trabecular separation than 30d-Sham rats. There was a main effect of both time (p = 0.0083) and condition (p = 0.0469) for trabecular number, but no interaction effect (p = 0.9166). 30d-Sham rats had greater trabecular number than 180d-SCI rats, but were not statistically different from 30d-SCI or 180d-Sham rats (Table 2).

### 3.4 Young female rats had a main effect of condition in trabecular bone volume at the proximal humerus, but only time-related changes (30d vs. 180d) at the proximal tibia

**Fig. 2:**
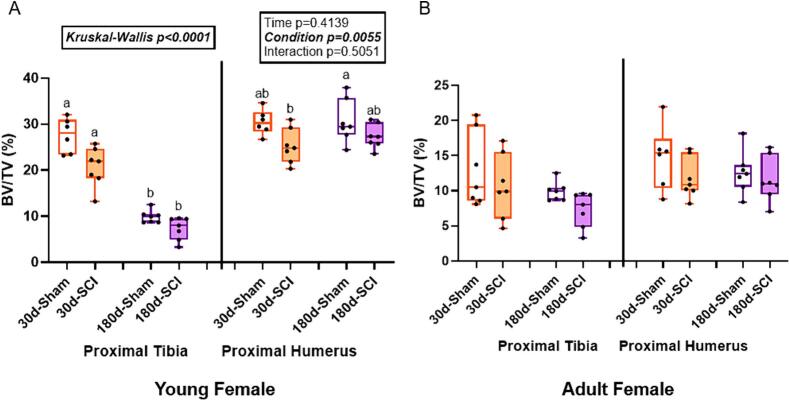
Trabecular bone volume (BV/TV, %) of sham and SCI rats at 30- and 180-days post injury in the proximal tibia (PT) and proximal humerus (PH) in young and adult female rats. A) In young females, there were not statistical differences between time-matched SCI and sham at the PT, but both 180d groups were lower than 30d groups. At the PH, there was an effect of condition, but SCI groups were not statistically different from their age-matched sham counterparts. B) In the adult females, there were no statistical differences at either the PT or the PH. n = 6–8/group. Groups not sharing the same letter are statistically different from each other within bone site. Main and interaction effect p-values from the 2 × 2 ANOVA are denoted in the box above the bone site. Data not normally distributed show the Kruskal-Wallis p-value in the box above the bone site.

At the proximal tibia in young females, trabecular bone volume did not meet normality assumptions (Kruskal-Wallis p < 0.0001). Both 30d groups had greater trabecular bone volume than both 180d groups (Fig. 2). There was a main effect of time (p = 0.0041) for trabecular thickness, but no effect of condition (p = 0.0695) or an interaction effect (p = 0.1183). Both 180d groups had thicker trabecular bone than 30d-SCI rats, but were not statistically different from 30d-Sham rats. Trabecular separation did not meet normality assumptions (Kruskal-Wallis p = 0.0001). Both 180d groups had greater trabecular separation than both 30d groups. Trabecular number did not meet normality assumptions (Kruskal-Wallis p < 0.0001). Both 30d groups had greater trabecular number than either 180d groups with 180d-Sham rats having greater trabecular number than 180d-SCI rats (Table 1).

At the proximal humerus in young females, there was a main effect of condition (p = 0.0055) for trabecular bone volume, but there was no effect of time (p = 0.4139) and no interaction effect (p = 0.5015). 180d-Sham rats had greater trabecular volume than 30d-SCI rats, but were not statistically different from 30d-Sham or 180d-SCI rats (Fig. 2). There was a main effect of time (p = 0.0335) for trabecular thickness, but no effect of condition (p = 0.0657) or interaction effect (p = 0.1375). 180d-Sham rats had thicker trabecular bone than 30d-SCI rats, but were not statistically different from either 30d-Sham or 180d-SCI rats. Trabecular separation did not meet normality assumptions (Kruskal-Wallis p = 0.3051), but showed no differences. There were also no statistical differences in trabecular number at this bone site (p = 0.1720; Table 1).

At the L4 vertebra in young females, there was a main effect of both time (p = 0.0018) and condition (p = 0.0325), but no interaction effect (p = 0.5483). Both 180d groups had greater trabecular bone volume than 30d-SCI rats, but were not statistically different from 30d-Sham. There was a main effect of both time (p < 0.0001) and condition (p = 0.0042) for trabecular thickness, but no interaction effect (p = 0.3130). 30d-SCI rats had lower trabecular thickness than all other groups. The statistical model did not show significant differences for trabecular separation (p = 0.1339) or trabecular number (p = 0.3491; Table 2).

### 3.5 Adult female rats showed no statistical differences in trabecular bone volume at either the sublesional tibia or the supralesional humerus

At the proximal tibia in adult females, trabecular bone volume did not meet normality assumptions (Kruskal-Wallis p = 0.1146), but showed no statistical differences (Fig. 2). There was a main effect of time (p = 0.0057) for trabecular thickness, but no effect of condition (p = 0.5911) or interaction effect (p = 0.7619) and the Tukey post-hoc showed no statistical pairwise comparisons. The statistical model did not show statistical differences for trabecular separation (p = 0.0800) or trabecular number (p = 0.1466) at the proximal tibia in adult females (Table 1).

There was no effect of age or injury at the proximal humerus in adult females. The statistical model did not show significant differences for trabecular bone volume (p = 0.3659), trabecular thickness (p = 0.1428), trabecular separation (Kruskal-Wallis p = 0.3119), or trabecular number (p = 0.2428; Fig. 2, Table 1).

At the L4 vertebra in adult females, the statistical model also did not show significant differences for trabecular bone volume (p = 0.7934), trabecular thickness (p = 0.4678), trabecular separation (p = 0.3142), or trabecular number (p = 0.5734; Table 2).

### 3.6 Young males showed effects of time (30d vs. 180d) on cortical bone parameters, but no effects of SCI

At the midshaft tibia in young males, the statistical model did not show significant differences for total area (p = 0.1617). There was a main effect of time (p < 0.0001) and condition (p = 0.0123) for cortical bone area, but there was no interaction effect (p = 0.9578). Both 180d groups had greater cortical area than both 30d groups. There was a main effect of both time (p < 0.0001) and condition (p = 0.0039) for cortical thickness, but no interaction effect (p = 0.9354). Both 180d groups had greater cortical thickness than both 30d groups. There was a main effect of time (*p* < 0.0001) for cortical area fraction (CA/TA), but no effect of condition (p = 0.1577) or interaction effect (p = 0.2899). Similarly, 180d groups had greater cortical area fraction than either 30d group (Table 3).

**Table 3:** Cortical bone parameters. Total area (TA), cortical bone area (Ct.Ar), cortical area fraction (CA/TA), cortical thickness (Ct.Th). Groups not sharing the same letter within sex, age, and bone site are statistically different (p < 0.05).

	Site	Time	Condition	TA (mm^2^)	Ct.Ar (mm^2^)	CA/TA (%)	Ct.Th (mm)
Young Male	Tibia	30d	Sham	12.08 ± 0.50	5.72 ± 0.18^b^	0.47 ± 0.02^b^	0.42 ± 0.01^b^
			Contusion	11.08 ± 0.93	5.32 ± 0.28^b^	0.45 ± 0.02^b^	0.39 ± 0.01^b^
		180d	Sham	12.80 ± 0.95	6.81 ± 0.50^a^	0.53 ± 0.02^a^	0.49 ± 0.02^a^
			Contusion	12.16 ± 0.83	6.43 ± 0.43^a^	0.53 ± 0.02^a^	0.46 ± 0.04^a^
	Humerus	30d	Sham	7.67 ± 0.34^b^	4.35 ± 0.10^b^	0.57 ± 0.02	0.34 ± 0.02^b^
			Contusion	7.66 ± 0.47^b^	4.39 ± 0.13^b^	0.57 ± 0.03	0.34 ± 0.01^b^
		180d	Sham	8.75 ± 0.65^a^	5.14 ± 0.52^a^	0.59 ± 0.04	0.45 ± 0.03^a^
			Contusion	8.72 ± 0.83^a^	5.15 ± 0.16^a^	0.59 ± 0.04	0.46 ± 0.02^a^
Adult Male	Tibia	30d	Sham	12.56 ± 1.18	6.98 ± 0.42	0.56 ± 0.03	0.50 ± 0.02
			Contusion	12.55 ± 1.36	6.97 ± 0.29	0.56 ± 0.06	0.50 ± 0.02
		180d	Sham	11.79 ± 1.19	6.67 ± 0.44	0.56 ± 0.06	0.48 ± 0.03
			Contusion	13.36 ± 0.85	7.05 ± 0.39	0.53 ± 0.02	0.49 ± 0.01
	Humerus	30d	Sham	8.71 ± 0.85	4.69 ± 0.29^b^	0.44 ± 0.01^b^	0.54 ± 0.03
			Contusion	8.49 ± 0.59	4.77 ± 0.18^b^	0.45 ± 0.02^ab^	0.56 ± 0.02
		180d	Sham	8.50 ± 0.46	5.09 ± 0.33^ab^	0.60 ± 0.03^a^	0.46 ± 0.04
			Contusion	9.44 ± 0.877	5.57 ± 0.40^a^	0.59 ± 0.03^a^	0.47 ± 0.02
Young Female	Tibia	30d	Sham	9.23 ± 0.47	5.29 ± 0.18	0.57 ± 0.03	0.43 ± 0.04
			Contusion	8.83 ± 0.46	4.96 ± 0.26	0.56 ± 0.02	0.43 ± 0.03
		180d	Sham	9.07 ± 0.60	5.27 ± 0.26	0.58 ± 0.03	0.43 ± 0.03
			Contusion	8.82 ± 0.67	4.92 ± 0.29	0.56 ± 0.03	0.41 ± 0.03
	Humerus	30d	Sham	5.80 ± 0.12	3.72 ± 0.20^b^	0.64 ± 0.04^b^	0.42 ± 0.02^b^
			Contusion	5.60 ± 0.18	3.69 ± 0.14^b^	0.66 ± 0.02^b^	0.42 ± 0.02^b^
		180d	Sham	5.91 ± 0.27	4.35 ± 0.16^a^	0.73 ± 0.02^a^	0.46 ± 0.03^a^
			Contusion	5.94 ± 0.39	4.43 ± 0.20^a^	0.75 ± 0.02^a^	0.48 ± 0.02^a^
Adult Female	Tibia	30d	Sham	8.92 ± 0.64	5.14 ± 0.28^a^	0.58 ± 0.03	0.46 ± 0.03
			Contusion	8.42 ± 0.98	4.62 ± 0.28^b^	0.55 ± 0.03	0.43 ± 0.02
		180d	Sham	8.97 ± 0.98	4.92 ± 0.34^a^	0.55 ± 0.05	0.43 ± 0.03
			Contusion	8.76 ± 0.70	4.78 ± 0.22^a^	0.55 ± 0.03	0.43 ± 0.02
	Humerus	30d	Sham	5.97 ± 0.38	3.74 ± 0.14^ab^	0.42 ± 0.02	0.63 ± 0.04
			Contusion	5.59 ± 0.58	3.62 ± 0.21^b^	0.42 ± 0.02	0.63 ± 0.04
		180d	Sham	6.21 ± 0.50	4.07 ± 0.36^a^	0.66 ± 0.04	0.43 ± 0.03
			Contusion	6.15 ± 0.34	4.04 ± 0.15^a^	0.66 ± 0.03	0.44 ± 0.02

At the midshaft humerus in young males, there was a main effect of time (p = 0.0001) for total bone area, but no effect of condition (p = 0.9419) or interaction effect (p = 0.9804). Both 180d groups had greater total bone area than both 30d groups. Cortical bone area did not meet normality assumptions (Kruskal-Wallis p = 0.0004). Both 180d groups had greater cortical bone area than both 30d groups. Cortical thickness did not meet normality assumptions (Kruskal-Wallis p = 0.0003). Both 180d groups had greater cortical thickness than both 30d groups. The statistical model did not show significant differences for cortical area fraction (p = 0.4570; Table 3).

### 3.7 Adult male rats showed no differences in cortical bone parameters at the sublesional proximal tibia and only effects of time (30d vs. 180d) at the supralesional humerus

At the midshaft tibia in adult males, the statistical model did not show significant differences for total bone area (p = 0.1693), cortical bone area (p = 0.3520), cortical thickness (p = 0.5101), or cortical area fraction (p = 0.4932; Table 3).

At the midshaft humerus in adult males, the statistical model did not show significant differences for total bone area (p = 0.0841). There was a main effect of time (p < 0.0001) and condition (p = 0.0344) for cortical bone area, but no interaction effect (p = 0.1135). 180d-SCI rats had greater cortical bone area than both 30d groups, but were not statistically different from 180d-Sham rats. The statistical model did not show significant differences for cortical thickness (p = 0.0923). There was a main effect of time (p = 0.0050) for cortical area fraction, but no effect of condition (p = 0.5290) or interaction effect (p = 0.2199). Both 180d groups had greater cortical area fraction than 30d-Sham rats, but were not statistically different from 30d-SCI rats (Table 3).

### 3.8 In young female rats, there were also no effects of SCI on cortical bone parameters at the sub- or supralesional site

At the midshaft tibia in young females, the statistical model did not show significant differences for total bone area (p = 0.5054), cortical thickness (p = 0.4213), or cortical area fraction (p = 0.4910). There was a main effect of condition (p = 0.0022) in cortical bone area, but no effect of time (p = 0.7190) or interaction effect (p = 0.8865). The Tukey post-hoc showed no statistically different pairwise comparisons (Table 3).

At the midshaft humerus in young females, the statistical model did not show significant differences for total bone area (p = 0.0983). There was a main effect of time (p < 0.0001) for cortical bone area, but no effect of condition (p = 0.7574) and no interaction effect (p = 0.3775). Both 180d groups had greater cortical bone area than both 30d groups. There was a main effect of time (p < 0.0001) for cortical thickness but no effect of condition (p = 0.5262) and no interaction effect (p = 0.3362). Both 180d groups had greater cortical thickness than both 30d groups. There was a main effect of time (*p* < 0.0001) for cortical area fraction, but no effect of condition (p = 0.1964) and no interaction effect (p = 0.8688). Both 180d groups had greater cortical area fraction than both 30d groups (Table 3).

### 3.9 Adult female SCI rats had lower cortical bone area at the sublesional tibia, but no effect of condition (SCI vs. Sham) at the supralesional humerus

At the midshaft tibia in adult females, the statistical model did not show significant differences for total bone area (p = 0.6418), cortical thickness (p = 0.1340), or cortical area fraction (p = 0.4033). There was a main effect of condition (p = 0.0061) for cortical bone area, but no effect of time (p = 0.7433) or interaction effect (p = 0.0864). 30d-SCI rats had lower cortical bone area than all other groups (Table 3).

At the midshaft humerus in adult females, the statistical model did not show significant differences for total bone area (p = 0.2807), cortical thickness (p = 0.5420), or cortical area fraction (p = 0.3324). There was a main effect of time (p = 0.0004) for cortical bone area, but no effect of condition (p = 0.3881) or interaction effect (p = 0.6451). Both 180d groups had greater cortical bone area than 30d-SCI rats, but were not statistically different from 30d sham rats (Table 3).

### 3.10 Bone formation rate was lower in SCI rats at both the proximal tibia and the proximal humerus in young male rats

**Fig. 3:**
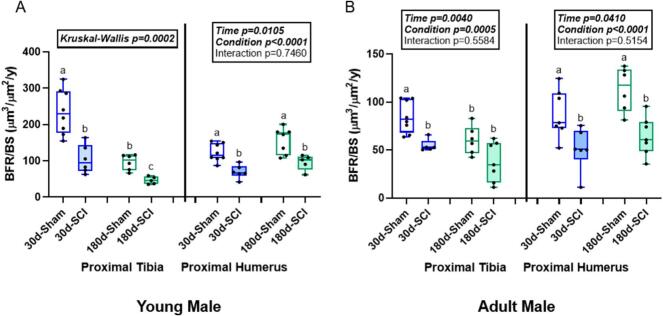
Bone formation rate (BFR/BS, μm^3^/μm^2^/y) at the proximal tibia (PT) and proximal humerus (PH) in male rats at 30- and 180-days post-injury. A) In young males, bone formation rate was lower in both SCI groups vs. age-matched sham at the PT. At the PH, both SCI groups had lower BFR than both sham groups. B) In adult males, 30d-SCI was lower than 30d-Sham at the PT. At the PH, both SCI groups were lower than both sham groups. n = 6–8/group. Groups not sharing the same letter are statistically different from each other within bone site. Main and interaction effect p-values from the 2 × 2 ANOVA are denoted in the box above the bone site. Data not normally distributed show the Kruskal-Wallis p-value in the box above the bone site.

At the proximal tibia in young males, there was a main effect of both time (p = 0.0009) and condition (p < 0.0001) for mineralized surface to bone surface (MS/BS), but no interaction effect (p = 0.0942). Both Sham rats had higher MS/BS than their time-matched SCI counterparts with 180d-Sham lower than 30d-Sham (Table 4). There was a main effect of both time (p < 0.0001) and condition (p = 0.0030) for mineralized apposition rate (MAR), but no interaction effect (p = 0.3604). 30d-Sham rats had greater MAR than 30d-SCI and both 180d groups (Table 4). 30d-SCI rats had greater MAR than 180d-SCI rats, but were not statistically different from 180d-Sham rats. Bone formation rate (BFR) did not meet normality assumptions (Kruskal-Wallis p = 0.0002). Both Sham groups had higher BFR than time-matched SCI groups with 180d-Sham lower than 30d-Sham (Fig. 3).

**Table 4:** Dynamic histomorphometry parameters. Mineralized surface to bone surface (MS/BS), mineral apposition rate (MAR). Groups not sharing the same letter within sex, age, and bone site are statistically different (p < 0.05).

	Site	Time	Condition	MS/BS (%)	MAR (μm)
Young Male	Tibia	30d	Sham	35.01 ± 3.45^a^	1.84 ± 0.45^a^
			Contusion	22.50 ± 2.81^b^	1.26 ± 0.38^b^
		180d	Sham	26.91 ± 4.35^b^	0.99 ± 0.18^bc^
			Contusion	19.45 ± 3.41^c^	0.66 ± 0.13^c^
	Humerus	30d	Sham	25.68 ± 3.96^a^	1.31 ± 0.16^a^
			Contusion	20.40 ± 1.96^b^	0.95 ± 0.28^b^
		180d	Sham	32.85 ± 4.06^a^	1.29 ± 0.26^ab^
			Contusion	22.26 ± 2.0^b^	1.19 ± 0.25^ab^
Adult Male	Tibia	30d	Sham	22.27 ± 3.48^a^	1.04 ± 0.11^ab^
			Contusion	13.80 ± 1.64^b^	1.10 ± 0.18^a^
		180d	Sham	19.67 ± 4.96^ab^	0.85 ± 0.11^b^
			Contusion	14.75 ± 6.79^b^	0.67 ± 0.12^b^
	Humerus	30d	Sham	16.37 ± 3.01b	1.46 ± 0.22
			Contusion	11.75 ± 4.05b	1.13 ± 0.29
		180d	Sham	22.85 ± 4.34a	1.37 ± 0.20
			Contusion	15.38 ± 5.03b	1.16 ± 0.18
Young Female	Tibia	30d	Sham	21.60 ± 1.77^a^	1.17 ± 0.16
			Contusion	16.58 ± 3.82^ab^	1.03 ± 0.17
		180d	Sham	15.36 ± 4.51^b^	1.09 ± 0.22
			Contusion	18.10 ± 3.54^ab^	1.02 ± 0.13
	Humerus	30d	Sham	28.81 ± 5.70^a^	1.59 ± 0.19^a^
			Contusion	18.87 ± 3.29^b^	0.96 ± 0.22^b^
		180d	Sham	10.83 ± 2.26^c^	1.07 ± 0.34^b^
			Contusion	16.87 ± 2.79^b^	1.19 ± 0.23^b^
Adult Female	Tibia	30d	Sham	15.91 ± 6.35	1.16 ± 0.29^a^
			Contusion	11.98 ± 2.79	0.72 ± 0.25^b^
		180d	Sham	13.32 ± 3.36	1.09 ± 0.22^ab^
			Contusion	12.11 ± 4.05	1.02 ± 0.13^ab^
	Humerus	30d	Sham	18.39 ± 3.23	1.41 ± 0.14^a^
			Contusion	13.91 ± 2.19	1.17 ± 0.42^ab^
		180d	Sham	13.23 ± 3.67	1.01 ± 0.22^b^
			Contusion	12.33 ± 3.80	1.13 ± 0.13^ab^

At the proximal humerus in young males, MS/BS did not meet normality assumptions (p = 0.0005). Both sham groups had higher MS/BS than both SCI groups (Table 4). There was a main effect of condition (p = 0.0166) for MAR, but no effect of time (p = 0.2295) or interaction effect (p = 0.1730). 30d-sham rats had a higher MAR than 30d-SCI rats, but were not statistically different from either 180d groups (Table 4). There was a main effect of time (p = 0.0015) and condition (p < 0.0001) for BFR, but no interaction effect (p = 0.7460). Both Sham groups had higher BFR than both SCI groups (Fig. 3).

### 3.11 In adult male SCI rats, bone formation rate was lower in the sublesional proximal tibia at 30d days and lower in the supralesional humerus at both 30d and 180d post-injury compared to age-matched sham groups

At the proximal tibia in adult males, there was a main effect of condition (p = 0.0018) for MS/BS, but no effect of time (p = 0.6688) or interaction effect (p = 0.3581). 30d-Sham rats had higher MS/BS than both SCI groups, but were not statistically different from 180d-Sham rats (Table 4). There was a main effect of time (p < 0.0001) and an interaction effect (p = 0.0329) for MAR, but there was no effect of condition (p = 0.2386). 30d-SCI rats had higher MAR than both 180d groups, but were not statistically different from 30d-Sham rats (Table 4). There was a main effect of time (p = 0.0040) and condition (p = 0.0005) for BFR, but no interaction effect (p = 0.5584). 30d-Sham rats had higher BFR than all other groups (Fig. 3).

At the proximal humerus in adult males, there was a main effect of both time (p = 0.0055) and condition (p = 0.0013) for MS/BS, but no interaction effect (p = 0.3931). 180d-Sham rats had higher MS/BS than all other groups (Table 4). There was a main effect of condition (p = 0.0059) for MAR, but no effect of time (p = 0.7351) or interaction effect (p = 0.5369). The Tukey post-hoc showed no statistically different pairwise comparisons (Table 4):. There was a main effect of both time (p = 0.0410) and condition (p < 0.0001) for BFR, but no interaction effect (p = 0.5154). Both 30d groups had higher BFR than both 180d groups (Fig. 3).

### 3.12 Young female SCI rats had lower bone formation rate at the proximal humerus 30d post-injury than age-matched sham, but higher bone formation rate than age-matched sham at the proximal tibia at 180d post-injury

**Fig. 4:**
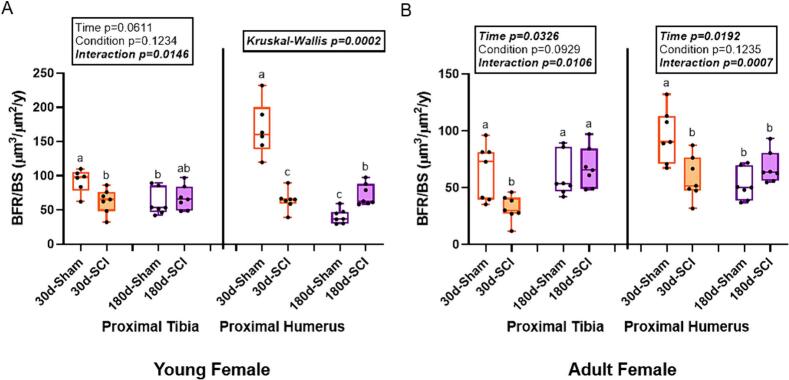
Bone formation rate (BFR/BS, μm^3^/μm^2^/y) at the proximal tibia (PT) and proximal humerus (PH) in adult female rats. A) In young female rats at the PTH, there was an interaction effect on bone formation rate with 30d-SCI lower than 30d-Sham, but the 180d groups not different from each other. At the PH, 30d-SCI was lower than 30d-Sham, but 180d-SCI was higher than 180d-Sham. B) In adult female rats, there was also an interaction effect with bone formation rate was lower in 30d-SCI vs. 30d-Sham, but both 180d groups not different from each other at the PT. At the PH, a similar trend was observed with 30d-SCI lower than 30d-Sham, but the 180d groups not different form each other at this site. n = 6–8/group. Groups not sharing the same letter are statistically different from each other within bone site. Main and interaction effect p-values from the 2 × 2 ANOVA are denoted in the box above the bone site. Data not normally distributed show the Kruskal-Wallis p-value in the box above the bone site.

At the proximal tibia in young females, there was an interaction effect (p = 0.0106) for MS/BS, but no effect of time (p = 0.1035) or condition (p = 0.4220). 30d-Sham rats had higher MS/BS than 180d-Sham rats, but were not statistically different from both SCI groups (Table 4). The statistical model did not show significant differences for MAR at the proximal tibia in young females (Table 4). There was an interaction effect (p = 0.0146) for BFR, but no effect of time (p = 0.0611) or condition (p = 0.1234). 30d-Sham rats had higher BFR than both 30d-SCI and 180d-Sham rats, but were not statistically different from 180d-SCI rats (Fig. 4).

At the proximal humerus in young females, there was both an effect of time (p < 0.0001) and an interaction effect (p < 0.0001) for MS/BS, but no effect of condition (p = 0.1641). 30d-Sham rats had greater MS/BS than 30-SCI while 180d-SCI had higher MS/BS than 180d-Sham (Table 4). There was a main effect of both condition (p = 0.0157) and an interaction effect (p = 0.0009) for MAR, but no effect of time (p = 0.1326). 30d-Sham rats had higher MAR than all other groups (Table 4). BFR did not meet normality assumptions (Kruskal-Wallis p = 0.0002). 30d-Sham rats had higher BFR than 30d-SCI while 180d-SCI rats had higher BFR than 180d-Sham rats (Fig. 4).

### 3.13 Adult female rats had time-by-condition interaction effects for BFR at both the sublesional tibia and supralesional humerus

At the proximal tibia in adult females, the statistical model did not show significant differences for MS/BS (p = 0.1121; Table 4). There was a main effect of both condition (p = 0.0076) and an interaction effect (p = 0.0479) for MAR, but no effect of time (p = 0.2169). 30d-Sham and 180d-SCI rats had a higher MAR than 30d-SCI rats, but were not statistically different from 180d-Sham rats (Table 4). There was a main effect of time (p = 0.0326) and interaction effect (p = 0.0106) for BFR, but no effect of condition (p = 0.0929). 30d-SCI rats had lower BFR than all other groups (Fig. 4).

At the proximal humerus in adult females, the statistical model did not show significant differences for MS/BS (p = 0.0854; Table 4). There was a main effect of time (p = 0.0310) for MAR, but no effect of condition (p = 0.5194) or interaction effect (p = 0.0701). 30d-Sham rats had higher MAR than 180d-Sham rats, but were not statistically different from both SCI groups (Table 4). There was a main effect of both time (p = 0.0192) and an interaction effect (p = 0.0007) for BFR, but no effect of condition (p = 0.1235). 30d-Sham rats had higher BFR than all other groups (Fig. 4).

### 3.14 Young male SCI rats had higher osteoclast-covered trabecular surfaces at the supralesional humerus at 30d post-injury compared to 30d-Sham

**Fig. 5:**
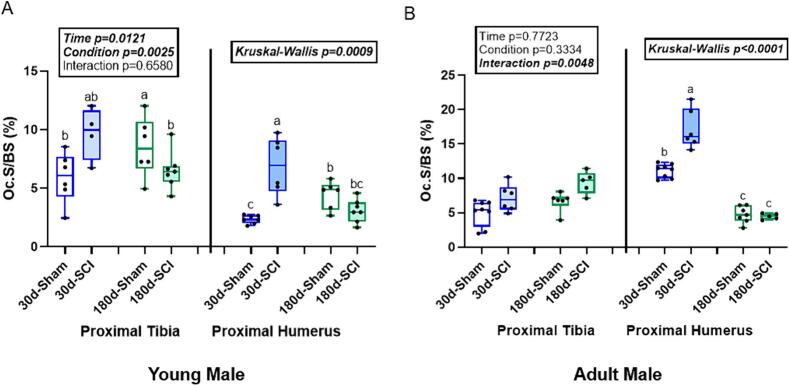
Osteoclast-covered trabecular surfaces (OcS/BS, %) at the proximal tibia (PT) and proximal humerus (PH) in young and adult male rats. A) In young male rats, there was an interaction effect, but no detectable differences in the post-hoc test at the PT. At the PH, 30d-SCI was higher than 30d-Sham with both 180d groups not statistically different from each other. B) In adult male rats at the PT, there was a main effect of condition, but both SCI groups were not statistically higher than their age-matched sham counterparts. At the PH, 30d-SCI was higher than 30d-Sham with both 180d-groups not different from each other. n = 6–8/group. Groups not sharing the same letter are statistically different from each other within bone site. Main and interaction effect p-values from the 2 × 2 ANOVA are denoted in the box above the bone site. Data not normally distributed show the Kruskal-Wallis p-value in the box above the bone site.

At the proximal tibia in young males, there was a main effect of both time (p = 0.0121) and condition (p = 0.0025) for osteoclast-covered surface/bone surface (OcS/BS), but there was no interaction effect (p = 0.6580). 180d-SCI had greater OcS/BS than 30d-Sham rats but were not statistically different from 30d-SCI or 180d-Sham rats (Fig. 5). At the proximal humerus in young males, OcS/BS did not meet normality assumptions (Kruskal-Wallis p < 0.0001). 30d-SCI rats had greater OcS/BS than all other groups while 30d-Sham rats had greater OcS/BS than both 180d groups (Fig. 5).

### 3.15 In adult male SCI rats, only the supralesional proximal humerus at 30d had higher osteoclast-covered surfaces compared to age-matched sham

At the proximal tibia in adult males, there was an interaction effect (p = 0.0048) for OcS/BS, but no effect of time (p = 0.7723) or condition (p = 0.3444). The Tukey post-hoc showed no statistically different pairwise comparisons (Fig. 5). At the proximal humerus in adult males, OcS/BS did not meet normality assumptions (Kruskal-Wallis p = 0.0009). 30d-SCI rats had greater OcS/BS than all other groups. 180d-Sham rats had greater OcS/BS than 30d-Sham, but were not statistically different from 180d-SCI rats (Fig. 5).

### 3.16 Osteoclast-covered surfaces in young female rats only showed effects of SCI at the supralesional humerus at the 30d post-injury timepoint with SCI higher than sham

**Fig. 6:**
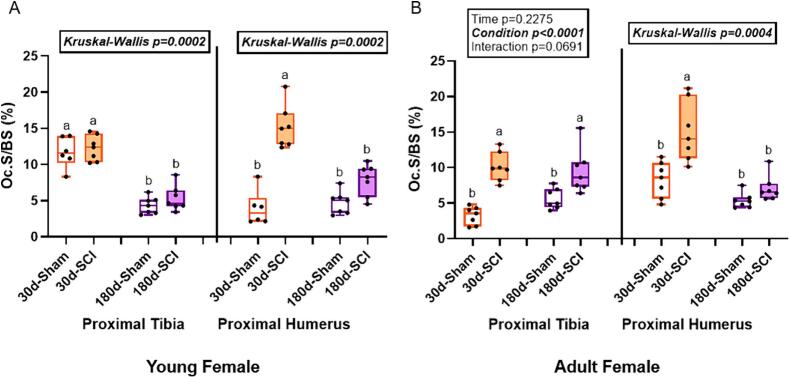
Osteoclast-covered trabecular surfaces (OcS/BS, %) at the proximal tibia (PT) and proximal humerus (PH) in young and adult female rats. A) In young female rats at the PT, both 180d groups were lower than both 30d groups. At the PH, 30d-SCI was higher than 30d-Sham with both 180d groups not different from each other. B) At the PT in adult female rats, both SCI groups had higher osteoclast-covered trabecular surfaces than both sham groups. At the PH, 30d-SCI was higher than 30d-Sham with both 180 groups not different from each other. n = 6–8/group. Groups not sharing the same letter are statistically different from each other within bone site. Main and interaction effect p-values from the 2 × 2 ANOVA are denoted in the box above the bone site. Data not normally distributed show the Kruskal-Wallis p-value in the box above the bone site.

At the proximal tibia in young females, OcS/BS did not meet normality assumptions (Kruskal-Wallis p = 0.0002). Both 30d groups had greater OcS/BS than both 180d groups with no difference between sham and SCI (Fig. 6). At the proximal humerus in young females, OcS/BS also did not meet normality assumptions (Kruskal-Wallis p = 0.0002). 30d-SCI rats had higher OcS/BS than all other groups (Fig. 6).

### 3.17 In adult female rats, SCI groups had higher osteoclast-covered trabecular surfaces at both 30d and 180d post-injury at the tibia, while the supralesional proximal humerus only showed higher OcS/BS in 30d-SCI rats

At the proximal tibia in adult females, there was a main effect of condition (p < 0.0001) for OcS/BS, but there was no effect of time (p = 0.2755) or interaction effect (p = 0.0691). Both SCI groups had greater OcS/BS than their age-matched sham counterparts (Fig. 6). At the proximal humerus in adult females, OcS/BS did not meet normality assumptions (Kruskal-Wallis p = 0.0004). 30d-SCI rats had higher OcS/BS than all other groups (Fig. 6).

## 4 Discussion

This preclinical study demonstrates that a moderate spinal cord contusion injury in rats leads to early alterations in bone turnover in long bones both below and above the spinal injury regardless of age or sex. This supports the concept of systemic factors having an influence on bone following SCI. After months of recovery, male SCI rats had continued suppression of bone formation rate at the supralesional humerus whereas female SCI rats had no differences compared to sham-treated counterparts. Therefore, sex-specific factors may influence the long-term response of bone following SCI.

Assessment of bone in SCI patients and animal models often focuses on the lower limb, due to high rates of fracture at this site. Moreover, the results of clinical studies that have looked at different skeletal sites are controversial. Some studies have shown that patients with SCI have lower bone mineral density (BMD) at sublesional sites, primarily the lower leg, with no differences in the distal forearm (Zehnder et al., 2004; Biering-Sørensen et al., 1990; Dauty et al., 2000; De Bruin et al., 2005; Ghasem-Zadeh et al., 2021b). Other studies have found lower bone density in the distal forearm of patients with paraplegia (Finsen et al., 1992; Sabo et al., 2001), but the difference in BMD between patients and controls was much greater in the lower limb (Finsen et al., 1992). Some differences have also been noted in the distal forearm between SCI patients and controls contingent on spinal injury level. For example, people living with paraplegia generally do not have changes in the distal forearm, a supralesional site, while those with quadriplegia/tetraplegia have bone loss at this site (Frey-Rindova et al., 2000; Demirel et al., 1998). However, one study with high resolution peripheral quantitative computed tomography showed no differences in the distal radius in both patients with paraplegia and quadriplegia (Ghasem-Zadeh et al., 2021b). Time post-injury also appears to affect the degree of supralesional bone loss. Clasey et al. reported that while SCI patients had normal-to-high BMD at the arm, there was an inverse relationship between BMD and time from injury at this site (Clasey et al., 2004). These data suggest a potential systemic effect of SCI that drives supralesional bone loss over time. At the lumbar vertebra, BMD has been reported as not different between SCI patients and control subjects (Garland et al., 2001; Sabo et al., 2001). However, it has also been noted that dual-energy x-ray absorptiometry (DXA) BMD measures of the lumbar spine in SCI patients are not accurate and quantitative computed tomography measures of BMD do show lower bone in SCI patients than controls (Liu et al., 2000). Taken together, these studies demonstrate that SCI causes significant loss of bone in the lower limb, but variable differences in bone at the lumbar spine and upper limb which likely reflects both the complexity of SCI-induced bone loss as well as the inherent challenges in controlling variables while studying this population.

In pre-clinical models, increased control over confounding variables, as well as weightbearing on four limbs enables more detailed understanding of the factors driving sublesional vs. supralesional bone alterations after SCI. Still, however, contradictory data are reported across studies. One study in female mice with a severe contusion injury found lower BMD at the tibia and femur by 1 week post injury and lower total body and humeral BMD by 4 weeks post injury (del Rivero and Bethea, 2016). Other studies have found no differences. In young male rats, there were not differences in humeral bone mineral content compared to controls 4 weeks after a complete lower thoracic spinal transection (Liu et al., 2008), while a separate study in rats found lower bone ash weight, but no difference in bone mineral content in the humerus and radius of young male rats 21 days after a lower thoracic spinal transection (Jiang et al., 2006). Differences in the length and type of injury likely could lead to these differing results.

In the current study, male SCI rats at the subchronic 30-day timepoint had ∼40 % lower trabecular bone volume at the sublesional tibia compared to age-matched sham rats with only a statistical difference in the adult rats. In the same male SCI rats, the trabecular bone volume of the humerus was 17–33 % lower than age-matched sham rats, statistically lower than shams at both ages. At the 180-day time point in male rats, trabecular bone volume was still 38–55 % lower in SCI vs. sham rats, but only statistically lower in the young males. Proximal humerus trabecular bone volume at was a non-significant 18 % lower in the young males, but only 9 % different between SCI and sham in the adult males. Cortical bone parameters were minimally affected at both the sub- and supralesional sites with most differences due to time and, therefore, age-related changes rather than SCI. Therefore, in male rats, subchronic changes in SCI appear to have effects leading to trabecular bone loss at both sublesional and supralesional long bonesites, albeit with a greater average difference in SCI bone vs sham bone at the sublesional tibia.

In the female rats, there were less differences at all sites than in male rats. While trabecular bone trended lower in SCI vs. Sham at both the proximal tibia and the proximal humerus in the young female rats, there were not statistical differences between the age-matched pairs. For adult females, there were no statistical differences at any site. Similar to the males, cortical bone changes were due to time and, therefore, age-related rather than SCI.

Interestingly, trabecular bone of the L4 vertebrae was less impacted in SCI rats than the trabecular bone of the long bones assessed. In young male and female rats, there were main effects of condition on trabecular bone volume (∼11–23 % lower than time-matched sham groups in males and 7–13 % lower than time-matched sham groups in females), but there were not statistically significant pairwise comparisons. In adult rats, there were not any statistical differences in trabecular bone volume at L4. Despite also being a sublesional site, the proximal tibia trabecular bone was more altered due to SCI. Interestingly, even the supralesional humerus showed more SCI-induced differences in trabecular bone volume than L4 in some ages and sexes. The reasons for the difference in sublesional bone loss is unclear, but it could be due to differences in bone marrow or neural signaling in long bones vs. vertebral bones. Additionally, the vertebral bone could respond differently to whatever circulating factors are influencing the trabecular bone of the proximal tibia and proximal humerus. Further, all rats in our study had the same T12 laminectomy and how this procedure may influence the bone of lumbar vertebrae is unknown.

Commensurate with the findings for bone volume in the sub- and supralesional long bones, all rats regardless of age or sex had lower bone formation rate at the sublesional proximal tibia and the supralesional proximal humerus at the 30-day post-injury time point. Additionally, at this early time point, osteoclast surfaces were higher in SCI humeri compared to sham in all ages and sexes. Again, the direction and magnitude of these changes in bone formation and osteoclast surfaces in the humeri were contrary to what we hypothesized. We hypothesized the supralesional bone, particularly at the earlier time point while rats were regaining lower limb locomotor function, would have minimal differences from sham or even greater bone formation due to increased/altered loading. Indeed, after 28 days in a hindlimb unloading model in rats, the weightbearing forelimbs had increased bone formation and decreased osteoclast surfaces, the opposite of the changes seen in the unloaded proximal tibia within the same rats (Metzger et al., 2020). Therefore, unloading without SCI resulted in location-specific bone alterations, while our current SCI rats had skeleton-wide alterations in bone turnover. This, again, alludes to the complexity of bone loss in SCI and points to the potential of systemic factors exerting influence on the skeleton post-SCI.

Interestingly, sex-based differences were apparent in bone turnover measures at the chronic recovery time point. Male rats of both ages maintained lower bone formation rate at the proximal humerus 180-days post-injury, but unlike the 30-day time point, there were not differences between sham and SCI in the humerus for osteoclast-covered trabecular surfaces. In the young female rats, the 180-day timepoint SCI humeri had higher bone formation rate than site-matched sham, while there were no statistical differences in the adult female rats. Likewise, osteoclast-covered trabecular surfaces were not different between sham and SCI at the proximal humerus at this chronic recovery time point. These changes point to potential increased recovery from SCI-induced bone changes in both the sub- and supralesional bone in females, while male rats generally maintained a lower bone formation rate at both skeletal sites despite the longer recovery time. Since both male and female rats had weightbearing of the forelimb throughout the entire study and months of weightbearing on the sublesional tibia at this extended recovery time point, these results allude to potential factors that lead to improved recovery from SCI-induced bone changes in a sex-dependent manner.

Few clinical and preclinical studies have assessed sex-based differences in bone following SCI (Raguindin et al., 2021). Yet, the number of women impacted by SCI is slowly increasing. Additional understanding of sex-based differences may reveal therapeutic options for preventing or reversing bone loss in females, irrespective of injury. In the clinical literature, some evidence points to greater loss of bone at the lower limb due to SCI in female vs. male counterparts (Garland et al., 2001) and higher fracture rates (Vestergaard et al., 1998) especially higher fracture rates in women over the age of 50 (Bethel et al., 2016). In our previous study, we found that, generally, both male and female rats at younger and middle-aged time points had tibial bone loss following a moderate contusion injury, but there were sex-specific differences particularly in bone turnover at different time points (Metzger et al., 2022). In the current study, clear sex-specific differences are seen, but only in the chronic recovery time point where male SCI rats maintained a reduction in bone formation rate at the proximal humerus, but female SCI rats had higher BFR (young) or not different BFR (adult) than their age-matched sham counterparts. In human studies, both low serum testosterone and low insulin-like growth factor-1 (IGF-1) have been noted in males with SCI (Bauman et al., 2006; Sullivan et al., 2017), both which could contribute to suppressed bone formation. Lower circulating testosterone has also been documented in male rats following SCI (Otzel et al., 2019). It is not clear what may be causing a systemic suppression in BFR in all sexes and ages at the earlier time point or how this response would be altered over time in only female rats; however, it seems likely a sex-specific factor, like testosterone, could be a driver of the prolonged suppressive effect in BFR in male rats at 180-days since the female rats in this study did not have similar outcomes.

Overall, treatments to protect bone after SCI have been only marginally successful, likely due to the complex nature of SCI-induced bone loss. In clinical studies with SCI patients, pharmacological treatments have generally been shown to be ineffective (Leone et al., 2023) and providers of SCI patients do not regularly prescribe FDA-approved medications for osteoporosis to their patients (Weaver et al., 2020). Non-pharmacological treatments like electrical stimulation with cycling have shown varying degrees of success at attenuating sublesional bone loss in SCI patients (Eser et al., 2003; Groah et al., 2010). However, to date, no activity-based physical therapy has been shown to completely prevent SCI-induced bone loss (Sutor et al., 2022). In animal models, electrical stimulation and activity-based therapies like bodyweight supported treadmill and isokinetic cycling have demonstrated attenuation of bone loss (Yarrow et al., 2023; Zhao et al., 2021). In our current study, the suppressed bone formation rate and increased bone resorption at the supralesional site in the earlier timepoint despite maintained regular loading throughout the duration of the study indicate that treatments focused only on the sublesional bone (loading, electrical stimulation of muscles, etc) may not be enough to counteract an SCI-induced systemic suppression in osteoblastic activity and elevated osteoclast surfaces. Likewise, systemic treatments (anti-resorptive or anabolic agents) may not be sufficient on their own to restore bone loss at the sublesional long bones which are at highest risk of fracture in the clinical population. Therefore, combination treatments that address both the local and systemic factors may be necessary to fully address SCI-induced bone alterations and reduce skeletal fragility in this population.

We can only speculate on what factors may be causing alterations in bone above the injury. The parent protocol of this study did assess serum inflammatory markers with the clearest sham vs. SCI differences in young female rats in IL-1β and IL-10 (Metzger et al., 2022). Interestingly, in our current assessment with these animals, the males had prolonged suppressed supralesional bone formation at the 180-day time point, but this finding does not correlate with inflammatory markers at the same time point. As mentioned above, SCI-induced alterations in endocrine factors like sex steroids, including estradiol^21,^and testosterone (Sullivan et al., 2017; Otzel et al., 2019), and IGF-I (Bauman et al., 2006) could also contribute to the altered supralesional bone turnover as well as the prolonged suppression of bone formation in male rats vs. female rats.

An important limitation of this study is the assessment of only a moderate contusion injury at one spinal level. The rats in this study did regain locomotor ability which, while showing the impact of SCI with return of regular loadbearing, may not correlate with more severe injuries where no locomotor ability is regained. How these results may translate to other degrees of contusion injury, vertebral levels of injury, or a transection model are unknown. Additionally, the data from middle-aged female rats in this study cannot be directly related to the clinical post-menopausal population as rats do not have the same cessation of estrogen production as humans do despite most of the middle-aged rats at 180-days being in constant estrus by study completion (Metzger et al., 2022). Therefore, the improved recoverability of bone in the female rats of both ages may not be relevant to the post-menopausal population.

In conclusion, this study demonstrates that SCI produces primarily short-term changes in bone homeostasis at both the sublesional tibia and supralesional humerus in both male and female rats. Importantly, one month following the injury, suppressed bone formation rate and increased osteoclast-covered surfaces was noted in SCI rats in both the sublesional tibia and supralesional humerus indicating SCI-induced alterations in bone turnover are not site-specific. After months of recovery, there were clear sex-based differences in the supralesional humerus, with continued suppression of bone formation rate in male SCI rats while female SCI rats had no differences compared to age-matched shams. Therefore, factors beyond site-specific alterations may lead to prolonged suppression of bone formation in male, but not female rats. Overall, these data continue to allude to the complex nature of bone loss in SCI and highlight the importance of therapeutics that address both local and systemic alterations in SCI.

## CRediT authorship contribution statement

**Corinne E. Metzger:** Conceptualization, Formal analysis, Investigation, Supervision, Visualization, Writing – original draft, Writing – review & editing. **Robert C. Moore:** Formal analysis, Investigation. **Landon Y. Tak:** Investigation. **Josephina Rau:** Investigation. **Jessica A. Bryan:** Investigation. **Alexander Stefanov:** Investigation. **Matthew R. Allen:** Conceptualization, Resources, Writing – review & editing. **Michelle A. Hook:** Conceptualization, Funding acquisition, Resources, Writing – review & editing.

## Declaration of competing interest

The authors have no conflicts of interest to declare.

## Data Availability

Data will be made available on request.

## References

[bb0005] Bauman W.A., Spungen A.M., Wang J., Pierson R.N., Schwartz E. (2006). Relationship of fat mass and serum estradiol with lower extremity bone in persons with chronic spinal cord injury. Am. J. Physiol. Endocrinol. Metab..

[bb0010] Bethel M. (2016). Risk factors for osteoporotic fractures in persons with spinal cord injuries and disorders. Osteoporos. Int..

[bb0015] Biering-Sørensen F., Bohr H.H., Schaadt O.P. (1990). Longitudinal study of bone mineral content in the lumbar spine, the forearm and the lower extremities after spinal cord injury. Eur. J. Clin. Investig..

[bb0020] Boehl G. (2022). Endocrinological and inflammatory markers in individuals with spinal cord injury: a systematic review and meta-analysis. Rev. Endocr. Metab. Disord..

[bb0025] Bouxsein M.L. (2010). Guidelines for assessment of bone microstructure in rodents using micro-computed tomography. J. Bone Miner. Res..

[bb0030] Carbone L.D. (2013). Morbidity following lower extremity fractures in men with spinal cord injury. Osteoporos. Int..

[bb0035] Clasey J.L., Janowiak A.L., Gater D.R. (2004). Relationship between regional bone density measurements and the time since injury in adults with spinal cord injuries. Arch. Phys. Med. Rehabil..

[bb0040] Comarr A.E., Hutchinson R.H., Bors E. (1962). Extremity fractures of patients with spinal cord injuries. Am. J. Surg..

[bb0045] Dauty M., Perrouin Verbe B., Maugars Y., Dubois C., Mathe J.F. (2000). Supralesional and sublesional bone mineral density in spinal cord-injured patients. Bone.

[bb0050] Davies A.L., Hayes K.C., Dekaban G.A. (2007). Clinical correlates of elevated serum concentrations of cytokines and autoantibodies in patients with spinal cord injury. Arch. Phys. Med. Rehabil..

[bb0055] De Bruin E.D., Vanwanseele B., Dambacher M.A., Dietz V., Stüssi E. (2005). Long-term changes in the tibia and radius bone mineral density following spinal cord injury. Spinal Cord.

[bb0060] Demirel G., Yilmax H., Paker N., Onel S. (1998). Osteoporosis after spinal cord injury. Spinal Cord.

[bb0065] Dempster D.W. (2013). Standardized nomenclature, symbols, and units for bone histomorphometry: a 2012 update of the report of the ASBMR Histomorphometry Nomenclature Committee. J. Bone Miner. Res..

[bb0070] Eser P. (2003). Effect of electrical stimulation-induced cycling on bone mineral density in spinal cord-injured patients. Eur. J. Clin. Investig..

[bb0075] Eser P. (2004). Relationship between the duration of paralysis and bone structure: a pQCT study of spinal cord injured individuals. Bone.

[bb0080] Finsen V., Indredavik B., Fougner K.J. (1992). Bone mineral and hormone status in paraplegics. Paraplegia.

[bb0085] Frey-Rindova P., De Bruin E.D., Stüssi E., Dambacher M.A., Dietz V. (2000). Bone mineral density in upper and lower extremities during 12 months after spinal cord injury measured by peripheral quantitative computed tomography. Spinal Cord.

[bb0090] Garland D.E., Adkins R.H., Stewart C.A., Ashford R., Vigil D. (2001). Regional osteoporosis in women who have a complete spinal cord injury. J. Bone Jt. Surg. - Ser. A.

[bb0095] Ghasem-Zadeh A. (2021). Heterogeneity in microstructural deterioration following spinal cord injury. Bone.

[bb0100] Ghasem-Zadeh A. (2021). Heterogeneity in microstructural deterioration following spinal cord injury. Bone.

[bb0105] Gibson A.E. (2008). C-reactive protein in adults with chronic spinal cord injury: increased chronic inflammation in tetraplegia vs paraplegia. Spinal Cord.

[bb0110] Gifre L. (2014). Incidence of skeletal fractures after traumatic spinal cord injury: a 10-year follow-up study. Clin. Rehabil..

[bb0115] Grassner L., Klein B., Maier D., Bühren V., Vogel M. (2018). Lower extremity fractures in patients with spinal cord injury characteristics, outcome and risk factors for non-unions. J. Spinal Cord Med..

[bb0120] Groah S.L., Lichy A.M., Libin A.V., Ljungberg I. (2010). Intensive electrical stimulation attenuates femoral bone loss in acute spinal cord injury. PM R.

[bb0125] Hayes K.C. (2002). Elevated serum titers of proinflammatory cytokines and CNS autoantibodies in patients with chronic spinal cord injury. J. Neurotrauma.

[bb0130] Hubscher C.H., Armstrong J.E., Johnson J.R. (2006). Effects of spinal cord injury on the rat estrous cycle. Brain Res..

[bb0135] Jiang S.D., Jiang L.S., Dai L.Y. (2006). Spinal cord injury causes more damage to bone mass, bone structure, biomechanical properties and bone metabolism than sciatic neurectomy in young rats. Osteoporos. Int..

[bb0140] Leone G.E., Shields D.C., Haque A., Banik N.L. (2023). Rehabilitation: neurogenic bone loss after spinal cord injury. Biomedicines.

[bb0145] Liu C.C. (2000). Quantitative computed tomography in the evaluation of spinal osteoporosis following spinal cord injury. Osteoporos. Int..

[bb0150] Liu D. (2008). Effects of spinal cord injury and hindlimb immobilization on sublesional and supralesional bones in young growing rats. Bone.

[bb0155] Maldonado-Bouchard S. (2016). Inflammation is increased with anxiety- and depression-like signs in a rat model of spinal cord injury. Brain Behav. Immun..

[bb0160] Metzger C.E., Gong S., Aceves M., Bloomfield S.A., Hook M.A. (2018). Osteocytes reflect a pro-inflammatory state following spinal cord injury in a rodent model. Bone.

[bb0165] Metzger C.E., Narayanan S.A., Phan P.H., Bloomfield S.A. (2020). Hindlimb unloading causes regional loading-dependent changes in osteocyte in fl ammatory cytokines that are modulated by exogenous irisin treatment. npj Microgravity.

[bb0170] Metzger C.E. (2022). Inflammaging and bone loss in a rat model of spinal cord injury. J. Neurotrauma.

[bb0175] Otzel D.M. (2019). Longitudinal examination of bone loss in male rats after moderate–severe contusion spinal cord injury. Calcif. Tissue Int..

[bb0180] Raguindin P.F., Muka T., Glisic M. (2021). Sex and gender gap in spinal cord injury research: focus on cardiometabolic diseases. A mini review. Maturitas.

[bb0185] del Rivero T., Bethea J.R. (2016). The effects of spinal cord injury on bone loss and dysregulation of the calcium/parathyroid hormone loop in mice. Osteoporos. Sarcopenia.

[bb0190] Sabo D. (2001). Osteoporosis in patients with paralysis after spinal cord injury. A cross sectional study in 46 male patients with dual-energy X-ray absorptiometry. Arch. Orthop. Trauma Surg..

[bb0195] Sinnott B. (2022). Risk factors and consequences of lower extremity fracture nonunions in veterans with spinal cord injury. JBMR Plus.

[bb0200] Sullivan S.D. (2017). Prevalence and etiology of hypogonadism in young men with chronic spinal cord injury: a cross-sectional analysis from two university-based rehabilitation centers. PM R.

[bb0205] Sutor T.W., Kura J., Mattingly A.J., Otzel D.M., Yarrow J.F. (2022). The effects of exercise and activity-based physical therapy on bone after spinal cord injury. Int. J. Mol. Sci..

[bb0210] Szollar S.M., Martin E.M.E., Parthemore J.G., Sartoris D.J., Deftos L.J. (1997). Densitometric patterns of spinal cord injury associated bone loss. Spinal Cord.

[bb0215] Vestergaard P., Krogh K., Rejnmark L., Mosekilde L. (1998). Fracture rates and risk factors for fractures in patients with spinal cord injury. Spinal Cord.

[bb0220] Wang T.D. (2007). Circulating levels of markers of inflammation and endothelial activation are increased in men with chronic spinal cord injury. J. Formos. Med. Assoc..

[bb0225] Weaver F.M. (2020). Spinal cord injury providers’ perspectives on managing sublesional osteoporosis. J. Spinal Cord Med..

[bb0230] Yarrow J.F. (2023). Passive cycle training promotes bone recovery after spinal cord injury without altering resting-state bone perfusion. Med. Sci. Sports Exerc..

[bb0235] Zehnder Y. (2004). Long-term changes in bone metabolism, bone mineral density, quantitative ultrasound parameters, and fracture incidence after spinal cord injury: a cross-sectional observational study in 100 paraplegic men. Osteoporos. Int..

[bb0240] Zhao W. (2021). Electrical stimulation of hindlimb skeletal muscle has beneficial effects on sublesional bone in a rat model of spinal cord injury. Bone.

